# Prenatal features of mandibulofacial dysostosis Guion-Almeida Type

**DOI:** 10.25122/jml-2020-0082

**Published:** 2021

**Authors:** Vlad Dragoi, Florina Nedelea, Nicolae Gica, Radu Botezatu, Gheorghe Peltecu, Anca Maria Panaitescu

**Affiliations:** 1. Obstetrics & Gynecology Department, Bucharest Emergency University Hospital, Bucharest, Romania; 2. Genetics Department, Filantropia Hospital, Bucharest, Romania; 3. Obstetrics & Gynecology Department, Carol Davila Medical University, Bucharest Romania; 4. Obstetrics & Gynecology Department, Filantropia Hospital, Bucharest, Romania

**Keywords:** mandibulofacial dysostosis, Guion-Almeida, whole exome sequencing, prenatal ultrasound

## Abstract

Facial dysostoses are clinically and genetically heterogeneous conditions characterized by congenital craniofacial anomalies which result from abnormal development of the first two pharyngeal arches and their derivatives during embryogenesis. Mandibulofacial dysostosis Guion-Almeida type (MFDGA) is a rare and relatively new syndrome described in the literature, first identified by Guion-Almeida *et al.* in 2000 and 2006. Another 108 cases have been documented after that. Prenatal diagnosis of this syndrome has not been described yet. Here we present the prenatal ultrasound findings in a case where MFDGA was confirmed after delivery. We suggest that MFDGA should be included in the prenatal differential diagnosis of syndromes with micrognathia and craniofacial anomalies.

## Introduction

Facial dysostoses are clinically and genetically heterogeneous conditions characterized by congenital craniofacial anomalies which result from abnormal development of the first two pharyngeal arches and their derivatives during embryogenesis. The pathology is then categorized by the association of a limb anomaly into mandibulofacial dysostosis and acrofacial dysostoses. A heterozygous mutation leading to the haploinsufficiency of EFTUD2, which encodes U5-116 kDa, a spliceosomal GTPase, is the cause behind the development of an MFDGA [1–4]. Mandibulofacial dysostosis Guion-Almeida type is a rare and relatively new syndrome described in the literature, first identified by Guion-Almeida *et al.* in 2000 and 2006 in 4 Brazilian children who presented zygomatic arch hypoplasia and accentuated micrognathia and associated severe language and speech delay [5, 6]. We present a case where abnormal ultrasound fetal features identified prenatally led to the post-partum diagnosis was of Guion-Almeida syndrome.

## Case Report

**Figure 1 AB.**
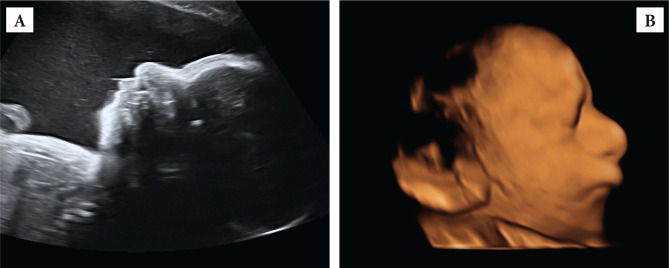
A – Ultrasound scan showing the abnormal fetal profile and polyhydramnios; B – 3D volumetric reconstruction of the fetal face showing atypical facial features and low set years.

A 31-years-old G1P1 woman was referred to our hospital at 33 weeks gestation for polyhydramnios and a small fetal stomach. She had a low risk for chromosomal abnormalities in the first trimester combined test and an unremarkable second-trimester ultrasound scan. Blood tests, including those for infections and gestational diabetes, were also normal. We confirmed the polyhydramnios (deepest vertical pool of 9) on fetal ultrasound examination and noted a small stomach, micrognathia, low set ears, increased prenasal thickness in an active fetus with normal growth, and Doppler velocimetry (Figure 1 AB). We counseled the couple on the possibility of oesophageal atresia, soft palate disorders, thoracic anomalies, and genetic syndromes. We discussed options, including invasive testing (amniocentesis) with fetal karyotyping, micro-array, and exome sequencing, which the couple declined. Because of the polyhydramnios and a reduced cervical length (1.5 cm), lung maturation with corticosteroid treatment was prescribed. An ultrasound re-evaluation took place 2 and 4 weeks later, showing a consistently growing fetus on the 10^th^ centile, with normal dopplers and the same previously mentioned elements. At 38 weeks gestation, labor induction was offered and undergone, and a 2600 g male baby was delivered with Apgar scores of 5 and 7 at 1 and 5 minutes. Clinical genetic examination described abnormal face shape, abnormality of the outer ear (preauricular skin tags), oesophageal atresia, choanal stenosis, cleft palate, microretrognathia, muscle weakness, tracheomalacia.

The differential diagnosis was considered in the first instance, Treacher-Collins syndrome or CHARGE syndrome. However, due to the complex clinical picture and phenotypic overlap between many genetic syndromes, the analysis of the Whole Exome was performed. This test allowed the simultaneous sequencing and analysis of all protein-coding regions (exons) of the genes in the human genome. Therefore, this approach increases the chances of finding the genetic cause in such difficult and rare phenotypes, in a shorter time, compared to the sequencing of individual genes or a small number of genes (panels).

In our case, the Whole Exome test was performed in an external laboratory. This test contains two main processes, respectively target-enrichment, and sequencing. Target enrichment is used in order to select and capture exomes from DNA samples. There are kits used for target enrichment. In our case, from the original report, Agilent's SureSelect Human All Exon V6 kit was used, and then the sequencing step on an Illumina platform. An average coverage depth of ~100x was achieved. Typically, ~97% of the targeted bases are covered >10x. An end-to-end in-house bioinformatics pipeline was applied, including base calling, alignment of reads to GRCh37/hg19 genome assembly, primary filtering of low-quality reads and probable artefacts, and subsequent annotation of variants. All disease-causing variants reported in HGMD®, ClinVar, or CentoMD®, and all variants with minor allele frequency (MAF) of less than 1% in the gnomAD database were considered. The evaluation was focused on coding exons along with flanking ±20 intronic bases. This analysis identified a heterozygous likely pathogenic variant c.2340_2341del p. (Cys780*) in the EFTUD2 gene. This variant creates a premature stop codon. This variant has also been confirmed by Sanger sequencing, according to guidelines. A genetic diagnosis of autosomal dominant Guion-Almeida type of mandibulofacial dysostosis was confirmed. To verify if this variant is inherited or “de novo” and establish a further recurrence risk, the genetic testing of the parents for the variant mentioned above was advised. The parents were informed about these implications, and they will have an informed choice in the future about testing. In Table 1 and Table 2, we determined the main characteristics of the Guion-Almeida syndrome highlighting in bold the prenatal ultrasound features and providing a differential diagnosis with syndromes with overlapping traits. These syndromes should all be considered in the differential diagnosis when prenatal features of craniofacial anomalies, including micrognathia, are encountered.

**Table 1.** Main characteristics of Guion-Almeida Syndrome; we highlight in bold those features that could potentially be diagnosed prenatally [2, 13].

**Guion-Almeida Syndrome**	
**Growth**	Short stature (suggested prenatally by the presence of IUGR)
**Head**	Microcephaly, trigonocephaly
**Face**	Midface hypoplasia, malar hypoplasia, prominent philtrum, micrognathia, buccal tags
**Ears**	Microtia, preauricular skin tags, external auditory meatus atresia, low-set ears, overfolded helices, hypoplasia of the upper part of the helix, dysplastic ears, conductive hearing loss
**Eyes**	Upslanting palpebral fissures, downslanting palpebral fissures, epicanthal folds, telecanthus
**Nose**	Choanal atresia (which results in breathing problems), upturned nose, short nose, anteverted nares
**Mouth**	Cleft palate (in some patients)
**Cardiovascular**	Heart: ASD, VSD (in some patients)
**Abdomen**	Esophageal atresia (in some patients) (suggested by the presence of polyhydramnios or an absent stomach echolucency)
**Skeletal**	Hands: preaxial polydactyly, slender fingers, proximally placed thumbs (in some patients)
**Neurologic**	CNS: delayed psychomotor development, severe speech delay, seizures (in some patients)

**Table 2.** Differential diagnosis with other syndromes [2, 4, 14–20].

**Syndrome**	**Common with Guion-Almeida**	**Particularities**	**Genes affected**
**Treacher Collins**	Malar hypoplasia (TCS1), microtia, choanal atresia, cleft palate (more often in TCS2 and TCS3), ear tags (TCS1).	Projection of scalp hair onto the lateral cheek (TCS1),zygomatic complex hypoplasia, mandibular hypoplasia; only downslanting palpebral fissures, lower eyelid coloboma, partial absence of lower eyelashes (TCS1); macrostomia (TCS1); motor and speech development delayed (some patients with TCS2).	TCOF1(TCS1); POLR1D(TCS2); POLR1C(TCS3).
**Nager**	Microcephaly, micrognathia, preauricular tags, low-set ears; cleft palate; VSD.	Retrognathia; partial or total absence of lower eyelashes, lower lid coloboma; cleft lip, macrostomia, trismus; Tetralogy of Fallot (in some patients); gastroschisis; syndactyly, clinodactyly.	SF3B4
**Diamond-Blackfan**	Micrognathia, midface hypoplasia; microtia; cleft palate(submucosal); delayed psychomotor development.	Only downslanting palpebral fissures, may have congenital diaphragmatic hernia; immobile thumbs at the interphalangeal joint; macrocytic anemia, increased fetal hemoglobin.	RPS28
**CHARGE**	Microcephaly, micrognathia, choanal atresia, cleft palate; esophageal atresia; VSD, ASD.	Lower ear abnormalities; cleft lip; Tetralogy of Fallot, DORV, pulmonary valve stenosis; duodenal atresia, anal atresia.	CHD7/SEMA3E
**Pierre Robin sequence**	Micrognathia, cleft palate	Glosoptosis	Genes related to SOX9

## Discussion

Mandibulofacial dysostosis Guion-Almeida type is a newly discovered and categorized entity with only 111 patients (including ours) reported in the literature for the past 18 years. Other cases were reported after 2006 by Ozkan *et al.*, Wieczorek *et al.*, and Lines *et al.* discovering additional signs of the genetic syndrome: oesophageal atresia, distal tracheoesophageal fistula, U-shaped cleft palate, low-set right ear with microtia, preauricular tags, choanal atresia, cleft palate, malar hypoplasia, delayed psychomotor development [7–9]. Lines *et al.* continued the work of the previously mentioned authors and identified seven patients with the same syndrome patterns: microcephaly, malar hypoplasia, most of them had preauricular tags, six infants had choanal atresia and cleft palate [3]. Vincent *et al.* encountered 11 genetically tested patients for the EFTUD2 gene, with mutations being found in 4 of them. All 4 cases had microcephaly, malar, and mandibular hypoplasia, and one presented oesophageal atresia [10]. A recent study by Yu *et al.* researched all the identified 108 cases of mandibulofacial dysostosis Guion-Almeida in the literature and added two new patients. Out of these 110 cases, 95 had molecular confirmation, and 28 had EFTUD2 splice site variants. 96.6 % of the patients presented dysplastic ear, accompanied by a hearing deficit (83.7%), 88% presented microcephaly, 31.3% had thumb anomalies, 31.5% cardiac defects, 26.1% oesophageal atresia/tracheoesophageal fistula [11]. The presence of accompanying seizures was reported by Vincent *et al.* and Matsuo *et al.* [10, 12]. As mentioned above, we have included in Table 1 and 2 main characteristics of the Guion-Almeida syndrome with prenatal ultrasound features and providing a differential diagnosis with syndromes with overlapping traits. These syndromes should all be considered in the differential diagnosis when prenatal features of craniofacial anomalies, including micrognathia, are encountered.

## Conclusion

The case we presented in this article is perhaps the only one where we describe the features of this genetic syndrome prenatally and confirm the diagnosis with a genome sequencing exam. We argue that MFDGA should be included in the differential diagnosis of micrognathia and other craniofacial anomalies diagnosed prenatally.

## Acknowledgments

### Conflict of interest

The authors declare that there is no conflict of interest.

### Consent for publication

A signed consent form was obtained for presenting this case.
